# The role of comorbid depressive symptoms on long-range temporal correlations in resting EEG in adults with ADHD

**DOI:** 10.1007/s00406-022-01452-2

**Published:** 2022-07-04

**Authors:** Jue Huang, Eike Ahlers, Holger Bogatsch, Pierre Böhme, Thomas Ethofer, Andreas J. Fallgatter, Jürgen Gallinat, Ulrich Hegerl, Isabella Heuser, Knut Hoffmann, Sarah Kittel-Schneider, Andreas Reif, Daniel Schöttle, Stefan Unterecker, Matti Gärtner, Maria Strauß

**Affiliations:** 1 Department of Psychiatry and Psychotherapy, University of Leipzig, 04103 Leipzig, Germany; 2 Department of Psychiatry and Psychotherapy, Charité – Universitätsmedizin, 10117 Berlin, Germany; 3 MSB Medical School Berlin, 14179 Berlin, Germany; 4 Clinical Trial Centre Leipzig, Faculty of Medicine, University of Leipzig, 04107 Leipzig, Germany; 5 Department of Psychiatry Psychotherapy and Preventive Medicine, University Hospital of Bochum, 44791 Bochum, Germany; 6 Department of Biomedical Magnetic Resonance, University Hospital of Tübingen, 72076 Tübingen, Germany; 7 Department of Psychiatry and Psychotherapy, Tübingen Center for Mental Health (TüCMH), University of Tübingen, 72076 Tübingen, Germany; 8 Department of Psychiatry and Psychotherapy, University Medical Center Hamburg-Eppendorf, 20251 Hamburg, Germany; 9 Department of Psychiatry, Psychotherapy and Psychosomatic Medicine, University Hospital of Frankfurt – Goethe University, 60528 Frankfurt am Main, Germany; 10 Department of Psychiatry, Psychosomatics and Psychotherapy, University Hospital of Würzburg, 97080 Würzburg, Germany

**Keywords:** Long-range temporal correlation, LRTC, ADHD, Depression, EEG, Resting state

## Abstract

Attention deficit/hyperactivity disorder (ADHD) is a neurodevelopmental disorder, characterized by core symptoms of inattention, hyperactivity and impulsivity. Comorbid depression is commonly observed in ADHD-patients. Psychostimulants are recommended as first-line treatment for ADHD. Aberrant long-range temporal correlations (LRTCs) of neuronal activities in resting-state are known to be associated with disorganized thinking and concentrating difficulties (typical in ADHD) and with maladaptive thinking (typical in depression). It has yet to be examined whether (1) LRTC occur in ADHD-patients, and if so, (2) whether LRTC might be a competent biomarker in ADHD comorbid with current depression and (3) how depression affects psychostimulant therapy of ADHD symptoms. The present study registered and compared LRTCs in different EEG frequency bands in 85 adults with ADHD between groups with (*n* = 28) and without (*n* = 57) additional depressive symptoms at baseline. Treatment-related changes in ADHD, depressive symptoms and LRTC were investigated in the whole population and within each group. Our results revealed significant LRTCs existed in all investigated frequency bands. There were, however, no significant LRTC-differences between ADHD-patients with and without depressive symptoms at baseline and no LRTC-changes following treatment. However, depressed ADHD patients did seem to benefit more from the therapy with psychostimulant based on self-report.

**Supplementary Information:**

The online version contains supplementary material available at 10.1007/s00406-022-01452-2.

## Introduction

Adult attention deficit/hyperactivity disorder (aADHD) is a frequent neurodevelopmental disorder with a worldwide prevalence of at least 2.8% [1]. It is characterized by dysfunctions in attention, behavior and emotion, in other words inattention (IA), hyperactivity (HY) and impulsivity (IMP) [2]. ADHD has a childhood onset and in about 60% of pediatric patients the symptoms persist into adulthood [3]. Comorbid psychiatric disorders are very commonly observed in aADHD [4] and can impact its persistence into adulthood [2].

In a range of studies, aADHD has been associated with co-occurrence of depressive disorder [5–7]. In self-evaluating assessments, depressed aADHD patients, when compared to patients had only ADHD, report a higher demand for previous mental health care [8], experience lower occupational functioning [9, 10] and perceive consequently lower quality of life [11]. Comorbid depression therefore presents important clinical challenges [12, 13] since its co-occurrence leads to greater illness burden and complexity than those individuals with aADHD or depression alone [11]. On the other hand, there is growing evidence from neuropsychological and electrophysiological studies that suggests no significant difference in cognitive performance [14] or in absolute power in specific frequency band measuring by electroencephalography (EEG) between depressed and non-depressed aADHD [15–18]. These results may question the usefulness and reliability of objective markers in differentiating between ADHD and depressive symptoms in adults. Several studies have furtherly concluded that EEG cannot be recommended as an appropriate diagnostic tool for ADHD based on the current level of knowledge [19], but still has a potentially promising future [20]. To sum up, despite those result discrepancies requiring clarifications, considering the greater burden and complexity of comorbid ADHD and depression, other suitable objective, diagnostic EEG-based markers are needed.

Neural oscillations arising from synchronized electrical activity of numerous neurons during resting state are not random but follow complex temporal structures [21]. It was well demonstrated that these neural oscillations are correlated over thousands of oscillation cycles [22–24]. This is a phenomenon called long-range temporal correlation (LRTC) and has been observed in several EEG studies [21, 22, 24]. In heathy individuals, the LRTC reflects the adaptability, i.e. the ability to maintain the balance between stability and flexibility of neuronal assemblies [25]. The utilization of LRTC during resting state as an indicator for adaptability of neural system involving behavior [24], sensory [26] and cognition [27–29] became recently evident. Aberrant LRTC has been observed in several psychiatric or neurological pathological conditions: LRTC was stronger in seizure-affected areas in epileptic [30] and depressive patients [31, 32]. While LRTC was weakened in early-stage of Alzheimer’s disease [33] and in patients with schizophrenia [34].

ADHD is associated with several neurocognitive deficits including difficulty in inhibiting non-beneficial behaviors, poor working-memory, concentration abilities and emotional instability. ADHD shares symptoms liking cognitive impairment in Schizophrenia [34], depression [31, 32] and Parkinson’s disease [35]. Given the evidence utilization of LRTC in mentioned disorders as well as its sensitivity to brain maturation in humans, it has been suggested as a potential biomarker of pathophysiology in neurodevelopmental disorders, in particular ADHD [36]. However, to the best of our knowledge, LRTC has not been investigated in ADHD yet.

For the treatment of aADHD, psychostimulants are recommended as first-line treatment as they have been shown to improve attention and enhance central nervous system (CNS) arousal [37]. Given the accumulating evidence supporting the availability of LRTC in treatment evaluation in a range of neurodevelopmental and mental disorders [32, 38, 39], it is important to investigate whether LRTC are modulated by means of treatment with psychostimulants in patients with aADHD.

Furthermore, several previous studies demonstrated significant associations between LRTC and severity of depressive symptoms in clinically depressed patients [40–43] and aberrations in LRTC of depressed patients compared to heathy controls A recent study also showed that the normalization of LRTC is associated with depressive symptoms relief [32].

Against this background, the main aims of this study were to examine the presence of LRTC in aADHD in resting EEG and to investigate whether LRTC are modulated by means of pharmacological intervention with methylphenidate in these patients. The second aim of this study was to explore whether LRTC is yet a competent biomarker of pathophysiology in aADHD with additional depressive symptoms, and if so, how and to which extend the effect of pharmacological intervention with extended release methylphenidate is modulated by comorbid depressive symptoms.

## Methods

### Participants

Data of the current study were taken from a previously published multicenter, single-arm, open label clinical trial in ADHD patients [44] with a recruitment period from April 2016 to June 2018. This study was reviewed and approved by the local ethics committee (Registration: EudraCT 2015–000,488–15; German Clinical Trial Register DRKS00009971, University of Leipzig ethics committee 337/15-ff). The methods utilized for patient inclusion and exclusion criteria have been described in detail elsewhere [44].

For the current analyses, patient data were included if they met the following criteria: clinical DSM-IV diagnosis of ADHD confirmed by a psychiatrist and psychologist, no evidence of current suicidality, anxiety or adjustment disorders, no history of substance abuse or dependence and no psychotic disorders. Patients also had to have completed a titration phase with extended release methylphenidate for 4 weeks. Exclusion criteria were demonstration of acute episodes of major depression according to ICD-10 during patient screening, pathological activity or excessive artifacts in resting EEG and remaining insufficient EEG epochs for LRTC analysis (see section EEG data analyses) at baseline or at the final visit, respectively.

### Study design and measurements

ADHD symptom severity: At baseline, a set of self-report measurements was used to evaluate ADHD-related symptoms: the short German Wender Utah Rating Scale (WURS-k) [45] was administered allowing a retrospective diagnosis of ADHD in childhood. WURS-k manifests excellent retest-reliability (*r* = 0.90) and internal consistency (*r* = 0.91), significant correlations were found to impulsivity in Eysenck’s Impulsivity Questionnaire, and excitability, aggression, emotional instability and satisfaction on the Freiburg Personality Inventory in ADHD patients [45]. The German version of the Conners’ Adult ADHD Rating Scale (CAARS) [46] was performed for a comprehensive assessment through three subscales consisting of DSM-IV ADHD symptoms (i.e. subscale DSM-IA, DSM-HYI and DSM-G) and four empirically derived subscales consisting of inattention/memory problem (IA/ME), emotional lability (IMP/EL), hyperactivity/restlessness (HY/RE), and problems with self-concept (SC). Internal consistency of these four subscales ranged between 0.87 and 0.88, furthermore they have probed to show very good fit confirmatory factor analysis with model for American original [47]. In addition, CAARS contains a scale of ADHD-Index. This index includes nine items that best distinguish between adults with ADHD and healthy controls. Sum score of WUSR-k as well as age- and sex-corrected T-scores for each subscale of the CAARS were further included into the analysis.

Depressive symptom severity and group distinction: Severity of depression was assessed using the German Beck Depression Inventory-II (BDI-II) [48]. Its sum score was included in further analysis. Group distinction between ADHD patients with and without additional depressive symptoms was based on the cut-off score in the BDI-II: Patients with a sum score over 13 at baseline indexed existence of at least a mild depression and were thus allocated into the ADHD + group; the remaining patients with sum scores in range of 1 to 13 were allocated into the ADHD- group. Sum score of Montgomery–Asberg Depression Rating Scale (MADRS) [49] were collected as an external assessment of the degree of depression.

Clinical related symptoms. Moreover, a set of questionnaires was employed to assess other psychological problems and status of each participant: the Severity Scale of the Clinical Global Impression (CGI-S) is a 7-point scale that requires clinician to rate the overall clinical severity of a participants’ illness at the time of assessment. The German Inventory of Interpersonal Problems (IIP) [50] identified participants’ most salient interpersonal difficulties. Subjectively perceived quality of life in different domains was measured with the short German Version of the World Health Organization Quality of Life Questionnaire (WHOQOL-BREF) [51].

Treatment. After diagnosis, all participants received 20 mg of extended release methylphenidate as initial dose. The dose was then increased weekly in steps of 20 mg until individual weight-based target doses (i.e. 40 mg/60 mg/80 mg per day) were reached. The dose could be adjusted or stopped due to any side effects at any time.

Reassessment. At the final visit, reassessment of all the above-mentioned instruments, except the WURS-k, allowed evaluation of the change from baseline due to the pharmacological intervention.

### EEG recording

To collect EEGs in eyes-closed resting condition, all participants were placed in a semi-supine position in a sound-attenuated and temperature-controlled room. They were instructed to rest with their eyes closed for a 15 min recording session. EEG was recorded from 31 Ag/AgCl active electrode positions according to the extended international 10–20 system using EasyCap (Brain Products GmbH, Gilching, Germany). Each EEG channel was referenced to a common average. Two bipolar electrodes monitored horizontal and vertical eye movements. Electrode impedance was kept below 10 kΩ.

### EEG data analyses

The EEG signal was analyzed in the Brain Vision Analyzer Software Version 2.1 (Brain Products GmbH, Gilching, Germany) and MATLAB (Version 2020a, The MathWorks, Inc., Natick, MA, USA). The data were first high pass-filtered at 1 Hz and down-sampled to 200 Hz. The first 10 min of the total 15 min EEG period were then selected in order to reduce the impact of different CNS arousal states on the LRTC in case participants had been falling asleep. Eye movements, muscle and cardiogenic artefacts were removed with an independent component analysis (ICA). After the ICA, the segmented EEG was recombined into continuous signals for subsequent analysis.

**Fig. 1:**
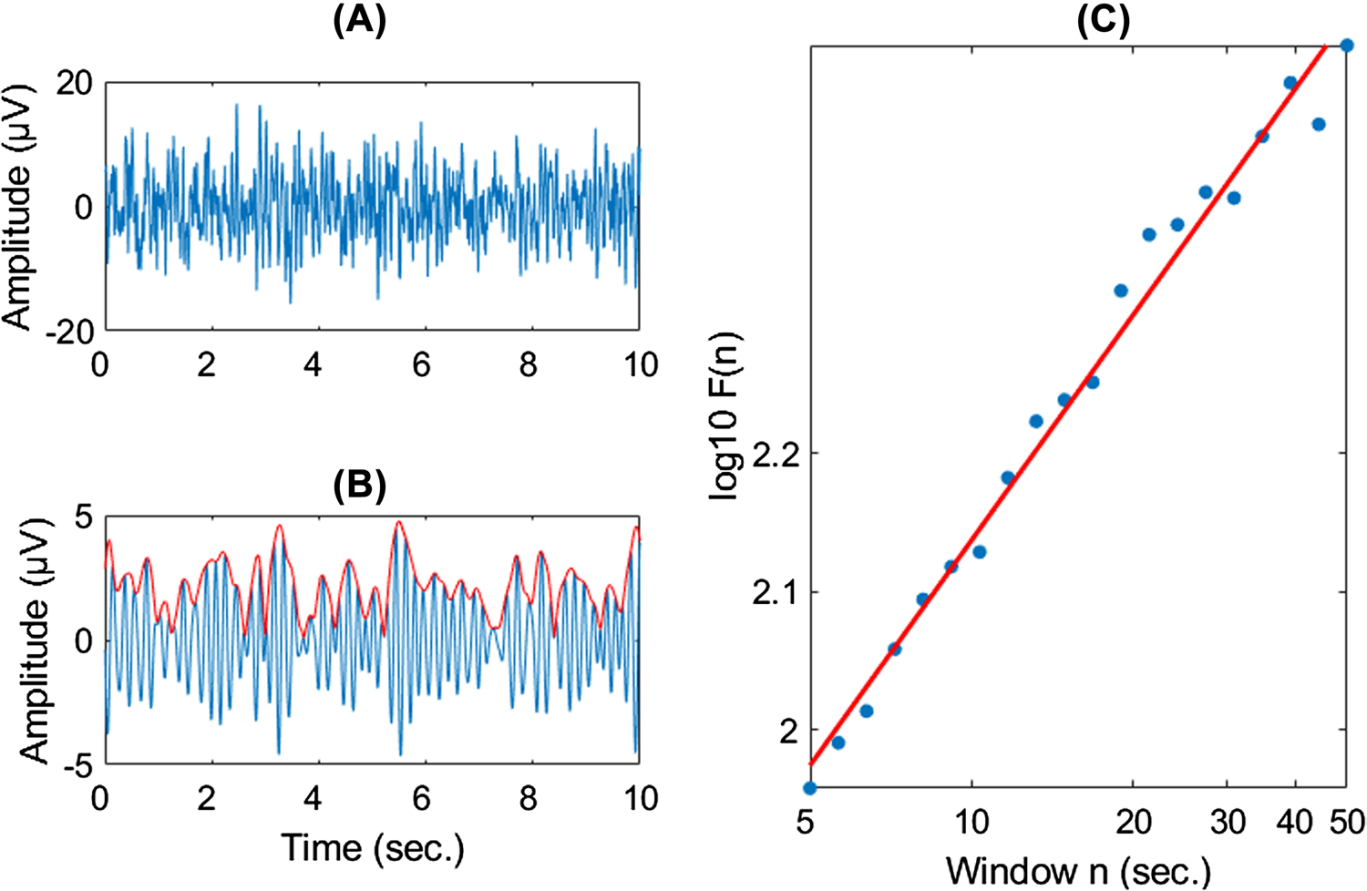
Stepwise explanation for estimating the scaling exponent of neural oscillation with detrended fluctuation analysis (DFA). **A** 10 s. of broadband EEG activity from Fz electrode in one patient with attention deficit/hyperactivity disorder. **B** Theta band (4–7 Hz) EEG activity (blue line) obtained from the signal in A using bandpass filtering and its amplitude envelope (red line) is extracted using Hilbert transform. **C** Mean fluctuation per window size in range 5–50 s. against on logarithmic axes. Scaling exponent is the slope of the best-fit line, in this case was 0.54

Afterwards, bandpass filtering was applied to filter signals in delta (1–3 Hz), theta (4–7 Hz), alpha (8–12 Hz) and beta (13–25 Hz) bands for all electrodes (an example signal of theta band at Fz site is illustrated in Fig. 1A). The Hilbert transformation was then applied to extract the amplitude envelope (red line in Fig. 1B) of the signals. The temporal structure of these amplitude envelopes was then analyzed using Detrended Fluctuation Analysis (DFA) [52] as implemented in a custom MATLAB script [32, 34]. DFA estimated the scaling of the root mean-square fluctuation of the intergraded and linearly detrended signal across different time windows [27], for the current study with a window size in range of 5–50 s. There was a nested artefact rejection function in DFA, with this the particular EEG segment exceeding an amplitude threshold ± 150 µV was marked as bad and ignored for further LRTC calculation. This step led to the exclusion of several participants for excessively bad EEG segments. The slopes of the least-squares lines were the scaling exponent (Fig. 1C) in a range of 0.5–1, where scaling exponent of 0.5 indicating for uncorrelated signals (e.g. white noise).

### Statistical analyses

In order to minimize the number of comparisons and correlational analyses, we calculated the mean scaling exponent through averaging scaling exponents at each electrode site in the corresponding frequency band (i.e. mean LRTC). In case of significant results, we conducted exploratory post hoc analyses for each single electrode. Statistical analyses were conducted using SPSS Statistics 25.0 (IBM corp.; Armonk, New York).

The relationship between LRTC and clinical symptoms was quantified by calculating Person product-moment correlation coefficient between LRTC in corresponding frequency band, depressive and ADHD symptoms.

Before we performed statistical comparisons, we executed Kolmogorov–Smirnov Tests to examine the normality of distribution of relevant variables. The results of Kolmogorov–Smirnov Tests are summarized (Table S1) in supplementary file. According to these results, non-parametric tests were applicable for age, CGI-S, BDI, MADRS, CAARS, WURS-K and LRTC, while parametric tests were for QoL and IIP.

In order to compare the LRTC between ADHD patients with (ADHD +) and without (ADHD-) additional depressive symptoms at baseline, Mann–Whitney-*U* Tests were performed. Baseline vs. Final comparisons of treatment related changes in LRTC were investigated by Wilcoxon Tests.

The correlational analyses and comparisons were done separately for LRTC in corresponding frequency band. Bonferroni adjusted alpha levels of 0.0125 per test was applied to avoid type I error inflation.

The differences of clinical characteristics at baseline between ADHD + and ADHD- was examined by independent sample *t*-Tests for normal distributed metric variables (QoL and IIP), while Mann–Whitney-*U* Tests were applied for non-normally distributed variables (age, CGI-S, BDI, MADRS, CAARS, WURS-K). Person Chi-Square Test were used for nominal variables (sex and dosage). The treatment related changes were investigated by paired-sample *t*-Tests/Wilcoxon Tests. Statistical significance value for these tests was set at 0.05.

Additionally, depending on the selected tests their corresponding between-group (ADHD + vs. ADHD-) and within-group (baseline vs. final) effect sizes (Cohen’s d or |r|) were calculated.

## Results

### Sample characteristics

The final sample consisted of 85 aADHD patients, who completed a titration phase with extended release methylphenidate for 4 weeks. There were 28 aADHD patients with additional depressive symptoms allocated into ADHD + group, while 57 aADHD patients without depressive symptoms in ADHD- group. This group distinction was based on the cut-off score (cut-off 13) in the BDI-II at baseline. Their main clinical characteristics are presented in Table 1.

**Table 1:** Sample description and comparisons of baseline clinical characteristics between ADHD patients with (ADHD +) and without (ADHD-) additional depressive symptoms as well as their changes due to intervention

		Baseline					Final visit					
		ADHD + (*n* = 28)	vs	ADHD-(*n* = 57)	*p*; effect size	All (*n* = 85)	ADHD + (*n* = 24)	vs. ADHS + at baseline^e^(*p*; effect size)	ADHD- (*n* = 47)	vs. ADHD- at baseline^e^(*p*; effect size)	All (*n* = 71)	vs. all at baseline^e^(*p*; effect size)
Females^a^		10 (35.7%)	vs	17 (29.8%)	.584; 0.059	27 (31.8%)						
Age (years)^b^		32.07 ± 7.40	vs	33.81 ± 10.12	.657; 0.048	33.24 ± 9.31						
Dosage^a^	40 mg	2 (7.1%)		2 (3.5%)	.606; 0.109	4 (4.7%)						
	60 mg	9 (32.1%)	vs	15 (26.3%)		24 (28.2%)						
	80 mg	17 (60.7%)		40 (70.2%)		57 (67.1%)						
CGI-S^b^		5.04 ± 0.33	vs	4.48 ± 0.99	**.001; 0.353**	4.67 ± 0.86	3.67 ± 0.64	**2.4E-5; 0.863**	3.25 ± 1.06	**1.2E-7; 0.781**	3.39 ± 0.96	**1.5E-11; 0.807**
BDI^b^		23.54 ± 8.03	vs	4.89 ± 3.94	**7.5E-14; 0.811**	11.04 ± 10.43	11.81 ± 10.87	**5.9E-5; 0.788**	4.56 ± 5.83	0.318; 0.149	7.21 ± 8.71	**9.0E-5; 0.464**
MADRS^b^		11.14 ± 3.26	vs	7.04 ± 3.49	**4.0E-6; 0.504**	8.42 ± 3.92	7.83 ± 4.56	**.023; 0.465**	5.31 ± 4.17	**0.007; 0.396**	6.15 ± 4.44	**4.4E-4; 0.417**
CAARS^b^	DSM-G	80.86 ± 10.15	vs	78.38 ± 9.44	0.166; 0.151	79.20 ± 9.69	64.54 ± 12.23	**4.0E-5; 0.839**	59.15 ± 12.68	**1.7E-8; 0.841**	61.00 ± 12.70	**3.2E-12; 0.839**
	DSM-IA	81.61 ± 10.74	vs	81.98 ± 8.34	0.797; 0.028	81.86 ± 9.15	66.13 ± 11.64	**9.6E-5; 0.796**	62.98 ± 15.86	**7.5E-8; 0.793**	64.04 ± 14.56	**2.8E-11; 0.795**
	DSM-HYI	74.46 ± 13.35	vs	68.55 ± 11.82	**0.042; 0.222**	70.52 ± 12.59	60.46 ± 11.90	**1.2E-4; 0.785**	55.15 ± 11.86	**1.7E-7; 0.772**	56.94 ± 12.06	**9.1E-11; 0.775**
	IA/ME	79.96 ± 10.49	vs	79.73 ± 9.63	0.864; 0.019	79.81 ± 9.86	65.54 ± 12.05	**3.0E-5; 0.852**	63.70 ± 16.36	**1.9E-7; 0.768**	64.32 ± 14.98	**3.2E-11; 0.793**
	HY/RE	73.14 ± 12.09	vs	70.25 ± 13.56	0.349; 0.102	71.21 ± 13.08	57.96 ± 12.65	**3.0E-5; 0.852**	52.11 ± 11.40	**1.4E-8; 0.837**	54.08 ± 12.07	**3.1E-12; 0.833**
	IMP/EL	73.86 ± 14.57	vs	63.64 ± 13.29	**0.002; 0.344**	67.05 ± 14.48	58.88 ± 13.46	**4.0E-5; 0.839**	50.98 ± 10.78	**7.8E-7; 0.728**	53.65 ± 12.25	**9.6E-11; 0.774**
	SC	71.96 ± 11.08	vs	64.13 ± 14.42	**0.002; 0.250**	66.74 ± 13.84	66.71 ± 10.27	**.009; 0.534**	51.94 ± 11.16	**1.3E-5; 0.642**	56.93 ± 12.88	**3.2E-7; 0.611**
	ADHS-Index	80.54 ± 8.92	vs	75.77 ± 8.56	**0.013; 0.271**	77.36 ± 8.92	64.79 ± 12.35	**1.5E-4; 0.773**	57.55 ± 13.11	**1.3E-8; 0.839**	60.00 ± 13.23	**8.7E-12; 0.816**
WURS-K^bc^		48.36 ± 11.46	vs	41.13 ± 11.41	**0.010; 0.282**	43.57 ± 11.86						
QoL^d^		205.12 ± 43.10	vs	258.75 ± 39.20	**1.52E-7; -1.324**	241.09 ± 47.59	235.24 ± 66.90	**0.012; -0.548**	277.77 ± 57.82	**0.009; -0.383**	263.20 ± 63.93	**2.67E-4; -0.412**
IIP^d^		119.54 ± 26.05	vs	87.67 ± 32.74	**2.2E-5; 1.037**	98.16 ± 34.06	99.35 ± 35.27	**0.004; 0.630**	75.81 ± 30.73	**0.007; 0.392**	83.54 ± 33.91	**8.60E-5; 0.442**

Entries are mean ± standard deviation or numbers (%)

Bold fonts indicate statistical significance; statistical significance value for these tests was set at 0.05

^a^Pearson Chi-Square Test for nominal variables, their effect sizes were estimate by Phi/Cramer’s V

^b^Mann–Whitney-Test for non-normally distributed variables, their effect sizes were estimate by |r|

^c^as a retrospective diagnosis of ADHD in childhood was only used at baseline

^d^Independent sample t-Tests for normally distributed variables, their effect sizes were estimate by Cohen’s d

^e^treatment related changes measuring by Wilcoxon Tests (effect size r), with exception of QoL und IIP. Their changes were measured by paired sample t-Tests (effect sizes Cohen’s d)

*CGI-S* Severity Scale of the Clinical Global Impression

*BDI* Beck Depression Inventory

*MADRS* Montgomery–Asberg Depression Rating Scale

*CAARS* the Conners’ Adult ADHD Rating Scale

*DSM-G* DSM-IA and DSM-HYI: three subscales of CAARS consisting DSM-IV ADHD symptoms

*IA/ME* inattention/memory

*HY/RE* hyperactivity/restlessness

*IMP/EL* impulsivity/ emotional lability

*SC* self-concept

*WURS-K* German Wender Utah Rating Scale

*QoL* quality of life as measured by German Version of the World Health Organization Quality of Life Questionnaire

*IIP* interpersonal problems as measured by German Inventory of Interpersonal Problems

Both groups are similar in sex, age and dosage. We obtained higher scores for individuals in the ADHD + group regarding depressive symptoms (MADRS) and clinical global impression (CGI-S) based on external assessment, than for those patients in the ADHD- group. The former also had significantly higher T-scores regarding currently ADHD severity as assessed by subjective rating (CAARS) and higher scores regarding ADHD severity in childhood based on subjective retrospective diagnosis (WURS-K). Furthermore, individuals in the ADHD + group as compared to those in the ADHD- group experienced more interpersonal difficulties (IIP) and lower quality of life (WHOQOL-BREF).

### LRTC in aADHD

Since the scaling exponent provided a quantitative measure for the LRTC of EEG signals, the scaling exponent in the 0.5–1.0 range indicates the presence of LRTC. According to this criterion, the mean LRTC was significantly present in the delta (mean = 0.61 ± 0.05, range: 0.54 − 0.81), theta (mean = 0.64 ± 0.08, range: 0.53 − 0.82), alpha (mean = 0.72 ± 0.08, range: 0.54 − 0.92) and beta (mean = 0.71 ± 0.08, range: 0.55 − 0.93) bands. The topographical distributions (Figure S1) and descriptive statistics at each electrode site (Table S2) are summarized in supplementary file.

### Mean LRTC in resting EEG are uncorrelated with ADHD and depressive symptoms

Table 2 shows detailed results for correlation analyses between mean LRTC and ADHD and depressive symptoms. There was a negative correlation between mean theta-LRTC and severity of depressive symptoms as measured by BDI sores (*r* =  – 0.228, *p* = 0.036) as well as between mean alpha-LRTC and T-score for subscale HY/RE of the CAARS (*r* =  – 0.243, *p* = 0.026), but they were not significant after Bonferroni correction (*p* ≤ 0.0125). No further significant associations were found between mean LRTC measured at resting EEG and T-scores obtained via CAARS and BDI scores (Table 2).

**Table 2:** Statistical results of correlation analyses between mean-LRTC and ADHD and depressive symptoms

Assessment and subscales		Mean delta-LRTC		Mean theta-LRTC		Mean alpha-LRTC		Mean beta-LRTC	
		*r*	*p*	*r*	*p*	*r*	*p*	*r*	*p*
BDI sum score		– 0.096	0.380	– 0.228	0.036	– 0.036	0.744	– 0.097	0.379
CAARS	DSM-G	0.138	0.210	0.099	0.372	– 0.032	0.771	0.087	0.433
	DSM-IA	0.170	0.123	0.188	0.087	0.116	0.292	0.197	0.073
	DSM-HYI	0.047	0.671	– 0.051	0.647	– 0.141	0.201	– 0.088	0.428
	IA/ME	0.088	0.425	0.124	0.260	0.063	0.566	0.059	0.592
	HY/RE	0.033	0.767	– 0.035	0.752	– 0.243	0.026	– 0.141	0.200
	IMP/EL	0.149	0.175	0.060	0.586	– 0.015	0.892	0.081	0.465
	SC	– 0.091	0.413	– 0.045	0.686	– 0.018	0.872	– 0.015	0.891
	ADHS-Index	– 0.032	0.772	– 0.012	0.916	– 0.041	0.712	0.042	0.705

*N* = 85

Statistical significance value for these tests was set at 0.0125

*BDI* beck depression inventory

*CAARS* the Conners’ adult ADHD rating scale

*DSM-G* DSM-IA and DSM-HYI: three subscales of CAARS consisting DSM-IV ADHD symptoms

*IA/ME* inattention/memory

*HY/RE* hyperactivity/restlessness

*IMP/EL* impulsivity/ emotional lability

*SC* self-concept

### No difference in mean LRTC at baseline between ADHD + and ADHD

**Fig. 2:**
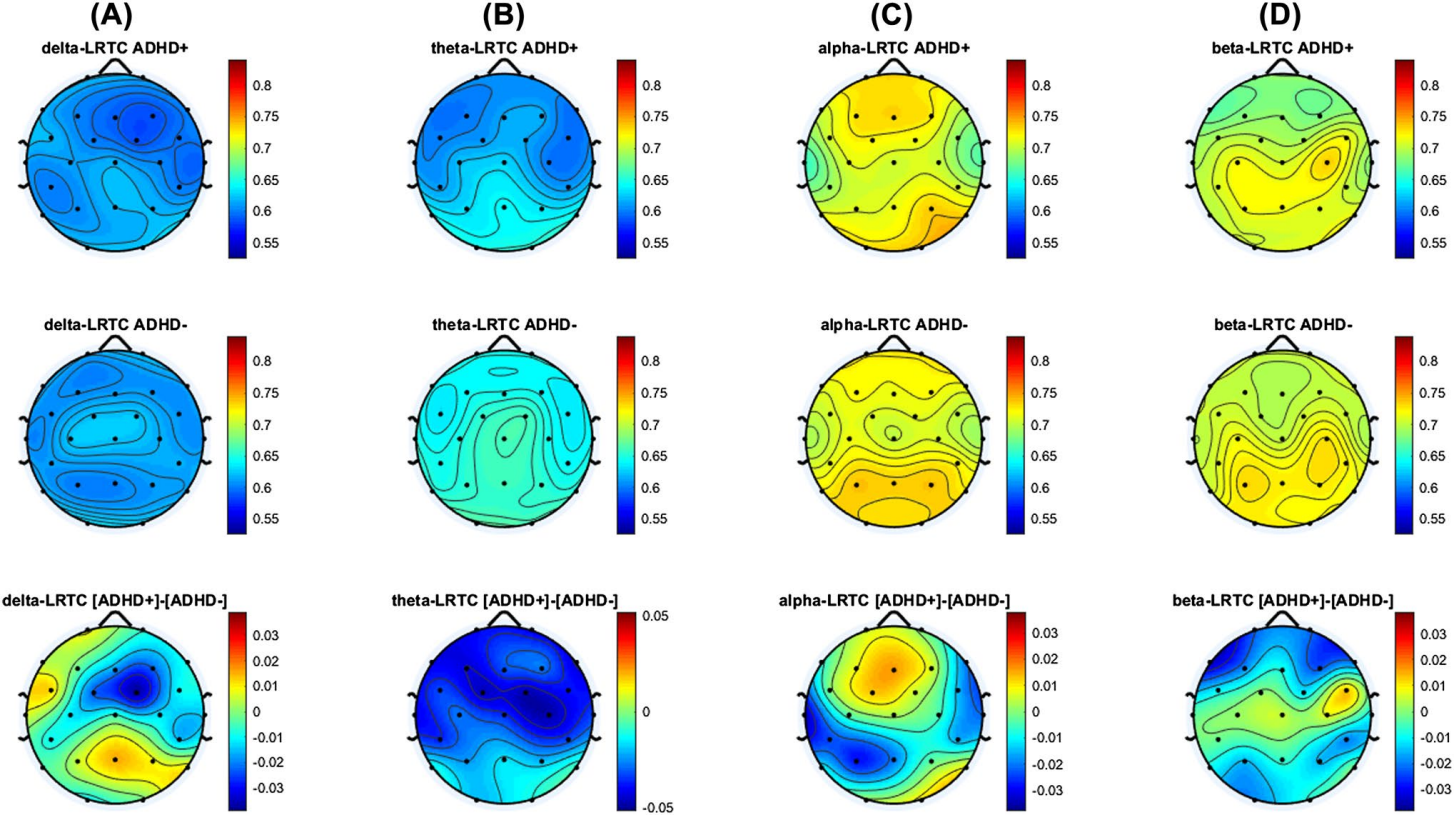
Topographical distributions of LRTC in delta (**A**), theta (**B**), alpha (**C**) and beta (**D**) oscillations in ADHD patients with (ADHD +) and without (ADHD-) depressive symptoms, the difference (ADHD + minus ADHD-) in corresponding frequency bands is presented in the bottom panel

Results of Mann–Whitney-U-Tests did not show any significant differences between ADHD + and ADHD- regarding mean LRTC in the delta (*p* = 0.695, |r|= 0.043), theta (*p* = 0.079, |r|= 0.191), alpha (*p* = 0.896, |r|= 0.097) or beta (*p* = 0.881, |r|= 0.016) bands. It is of note that a small effect size (|r|= 0.191) was found for the difference in mean theta-LRTC between ADHD + and ADHD-. Table 3 summarizes all related statistical results. The topographical distribution and comparisons between the groups at each electrode site are illustrated in Fig. 2.

**Table 3:** Comparisons of baseline mean LRTC between ADHD patients with (ADHD +) and without (ADHD-) depressive symptoms as well as their changes due to intervention

	Baseline					Final visit					
	ADHD + (*n* = 28)	vs	ADHD- (*n* = 57)	*p*^a^; effect size	All (*n* = 85)	ADHD + (*n* = 24)	vs. ADHD + at baseline^b^ (*p*; effect size)	ADHD—(*n* = 47)	vs. ADHD—at baseline^b^ (*p*; effect size)	All (*n* = 71)	vs. all at baseline^b^ (*p*; effect size)
Mean delta-LRTC	0.61 ± 0.04	vs	0.61 ± 0.05	0.695; 0.043	0.61 ± 0.05	0.61 ± 0.05	0.864; 0.035	0.63 ± 0.51	0.759; 0.045	0.62 ± 0.05	0.895; 0.014
Mean theta-LRTC	0.62 ± 0.06	vs	0.65 ± 0.08	0.079; 0.191	0.64 ± 0.08	0.61 ± 0.06	0.568: 0.117	0.64 ± 0.08	0.267; 0.162	0.63 ± 0.07	0.249; 0.125
Mean alpha-LRTC	0.72 ± 0.08	vs	0.71 ± 0.09	0.896; 0.097	0.72 ± 0.08	0.73 ± 0.11	0.627; 0.099	0.71 ± 0.10	0.824; 0.323	0.72 ± 0.11	0.954; 0.006
Mean beta-LRTC	0.70 ± 0.07	vs	0.71 ± 0.08	0.881; 0.016	0.71 ± 0.08	0.69 ± 0.09	0.424; 0.163	0.70 ± 0.10	0.138; 0.216	0.69 ± 0.09	0.095; 0.181

Entries are mean ± standard deviation

Statistical significance value for these tests was set at 0.0125

^a^comparisons between ADHD + and ADHD- using Mann–Whitney-*U*-Test

^b^treatment related changes measuring by Wilcoxon Tests

their effect sizes were estimate by |r|

### No change in mean LRTC due to pharmacological intervention

**Fig. 3:**
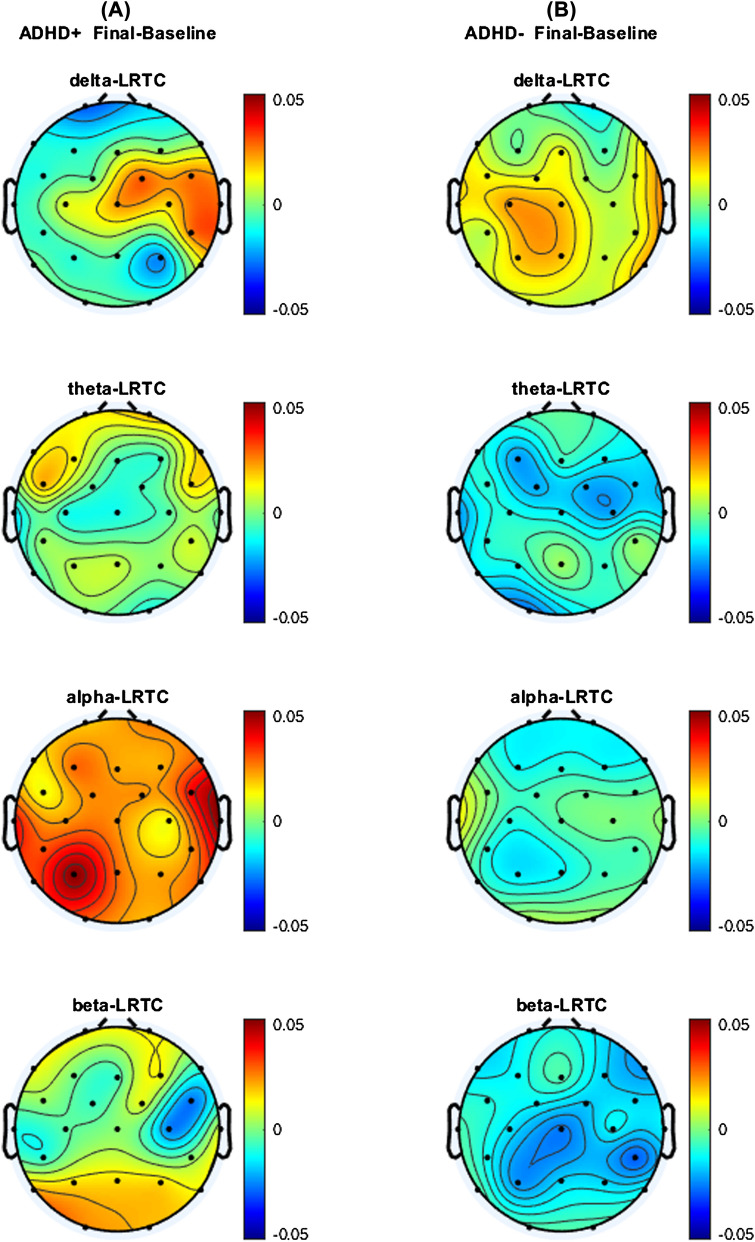
Topographical distributions of LRTC differences between the final visit and baseline in ADHD patients (**A**) with (ADHD +) and (**B**) without (ADHD-) additional depressive symptoms in the corresponding EEG frequency

After a 4-week intervention with extended release methylphenidate, no significant changes in mean LRTC in corresponding EEG frequency bands were observed in the entire population, nor within the ADHD + or ADHD- groups. Interestingly, the effect sizes for change due to intervention in mean delta-, theta- and beta-LRTC was slightly larger in the ADHD- than in the ADHD + group (delta of 0.045 vs. 0.035; theta of 0.162 vs. 0.117; alpha of 0.323 vs. 0.099; beta of 0.216 vs. 0.163). There was no clear indication that ADHD- and ADHD + group different regarding the LRTC-difference between baseline and final visit (supplementary file Table S3). The corresponding detailed statistical results are consolidated in Table 3. An overview of the topographical distribution and differences is provided in Fig. 3.

### The improvement in clinical symptoms due to pharmacological intervention

In the entire sample population, analyses of changes from baseline to final visits revealed a significant improvement in depressive (BDI, MADRS) and ADHD symptoms (CAARS). This improvement was in accordance with improvement in other clinical symptoms (CGI-S) as well as in interpersonal conflicts (IIP) and increased quality of life (WHOQOL-BREF) measured by questionnaires. Refer to Table 1 for a summary of the results.

The similar significant improvement was obtained in the ADHD + and the ADHD- groups: patients in the ADHD + group showed significant improvements in all investigated measurements. Likewise, patients in ADHD- group showed significant improvements in their existing symptoms, i.e. all investigated measurements except BDI. Patients in the ADHD + group had slightly larger effect sizes than patients in the ADHD- group for CGI-S (0.863 vs. 0.781), MADRS (0.465 vs. 0.396), IIP (0.630 vs. 0.392) and WHOQOL-BREF ( – 0.548 vs.  – 0.383). Additionally, effect sizes for differences in scores of CAARS subscale DSM-IA (0.796 vs. 0.793), DSM-HYI (0.785 vs. 0.772), IA/ME (0.852 vs. 0.768), HY/RE (0.852 vs. 0.837) and IMP/EL (0.839 vs. 0.728) between baseline and final visit were slightly larger in ADHD + than in ADHD- group. However, these two groups did no differ significantly regarding these differences between baseline and final visit (supplementary file Table S3).

## Discussion

The first aim of this study was to examine the presence of LRTC in adult patients with ADHD and to investigate their treatment-related changes. As a second aim of this study, we compared LRTC, severity of clinical symptoms and status between comorbidly depressed and non-depressed aADHD patients. We also investigated to which extend the effect of pharmacological interventions with psychostimulants was influenced by the additional depressive symptoms.

To the best of our knowledge, this is the first study that demonstrated the presence of LRTC in aADHD. Significant LRTCs were observed across all investigated EEG frequency bands. LRTCs are considered to reflect the developmental trajectories of human brains from childhood to adolescence, during adolescence and even up to early adulthood after which the temporal structure stabilizes [36]. Previous studies suggested that synaptic pruning extends well into adolescence [53, 54], and altered synaptic pruning is associated with those neurodevelopmental disorders that have prominent early onsets such as autism, ADHD and schizophrenia [55–57]. Moreover, the findings about attenuated LRTC in alpha and beta oscillations in schizophrenia [34, 58] and autism [59] indicated that there might be unusual rapid changes among different neural states in these patients, which was previously hypothesized to attribute to increased variabilities of neuronal activities [34]. Given the symptom overlaps between schizophrenia/autism and ADHD, it is reasonable to surmise that the aberration of the LRTC (very likely an attenuation) may as well be observed in aADHD when compared to healthy controls. This issue should be raised further in future studies.

Twenty-eight out of 85 of our aADHD patients reported additional depressive symptoms at baseline, which resulted in a prevalence of comorbid depression of about 33% in this study. Notably, this is not a representative prevalence since the patients with acute episodes of major depression according to ICD-10 criteria were already excluded during patient screening. Nevertheless, our aADHD patients reported depressive symptoms based on BDI-II. These patients did experience more difficulties in diverse interpersonal domains, perceived lower quality of life and showed worse self-concept as well as more emotional impulsivity (see Table 1) than those without depressive symptoms. Interestingly, in this study, depressed aADHD patients also reported severe ADHD symptoms already in childhood based on a subjective retrospective diagnosis (see Table 1), which implies a positive relationship between the severity of ADHD symptoms and the occurrence of comorbid major depression or further aggravation of ADHD-related features. These findings are supportive of results from a recent retrospective longitudinal study based on a German population, in which authors emphasized the positive influence of early recognition of ADHD on the prevention of development and aggravation of comorbid mental illnesses [60].

As above-discussed, our depressed aADHD showed further impairments due to the additional depressive symptoms, such further impairment on LRTCs is potentially reflected by the negative, but non-significant correlation between theta-LRTC and severity of depressive symptoms (*r* =  – 0.228, *p* = 0.036) and slight attenuated theta-LRTC in depressed aADHD with a small effect size (|r|= 0.191). Our findings, however, are in inconsistence with previous results reporting higher values of theta-LRTC in diagnosed depressed patients [32, 42]. In interpreting this discrepancy, it is important to mention that we excluded patients with moderate and severe depressive episode during patient screening. Our remaining aADHD patients, unlike typical depressive patients with increased persistence of maladaptive thinking, showed tendencies towards rapid mood changes and moment-to-moment dynamics in behavior due to emotional lability and hyperactivity/impulsivity, respectively.

In our entire sample population, there was an overall improvement in scores of all utilized ADHD symptom assessments at the final visit compared to the scores at baseline, after receiving extended release methylphenidate as monotherapy for 4 weeks (Table 1). These results suggest that the core symptoms of most of aADHD were reduced following treatment with methylphenidate with some of the patients (about 30%) even reaching therapy remission. The description and discussion of these results has already been published elsewhere [44].

In the present study, we mainly focused on the moderating effect of additional depressive symptoms on the treatment effect on ADHD symptoms. It is not surprising, that the majority of patients reported decreased depressive symptoms after treatment with methylphenidate, as the intake of methylphenidate has been shown to lead to amongst others increased extracellular dopamine levels [61], which has been proven a deficit in at least subgroups of depression [62]. Regarding the symptom comparison at baseline, our depressed aADHD presented more severe dysfunctions than non-depressed aADHD patients did. This remained the same at the final visit, with depressed aADHD patients still reporting more severe symptoms than those with ADHD only. This might indicate that depressed aADHD benefited from the treatment to the same extent as non-depressed aADHD did. This could be confirmed by the statistical results regarding the extent of changes in ADHD relevant symptoms (Table S3 supplementary file). However, the effect sizes for treatment induced symptom changes were slightly larger in global clinical impression (0.863 vs. 0.781) and depressive symptoms by external assessment (0.465 vs. 0.396), and were larger in interpersonal conflicts (0.630 vs. 0.392) and quality of life ( – 0.548 vs. -0.383) in our depressed aADHD patients. These findings implied that, though receiving the same treatment and same extents of changes in clinical symptoms, our depressed aADHD patients subjectively experienced more benefits from treatment with methylphenidate, above all in in domains of interpersonal relationships and quality of life.

In contrast to our expectation, no changes of LRTC were observed after 4-week monotherapy with methylphenidate (Fig. 3). Different from the result of this study, Gärtner et al. [32] demonstrated, after a brief treatment with either mindfulness meditation or stress reduction training for 2 weeks, a reduction of aberrant theta-LRTC in depressed patients. Similarly, through a close-loop stimulation neurofeedback treatment, alpha-LRTC was enhanced in healthy controls under resting conditions [63] or suppressed in patients with post-traumatic stress disorder [38]. On the other hand, in a sample on 21 non-comorbidly depressed aADHD patients, who received therapy with methylphenidate, the maintenance of normalization of EEG power normalization in theta band had been confirmed in a range from 2.5 to 9.7 months and its change in rest-to-task transition effects revealed to be moderately correlated with the dose of methylphenidate [64]. All these results convey a consensus: though there is preliminary evidence for the heritability [65] and high temporal stability of LRTC [22], its effect might possibly be achieved or registered by interventions acting wide distributed effect on brain or requiring joint coordination among different networks. Given evidence that the strength of LRTC increased while EEG power decreased [36], it would be of interest whether the theta-LRTC as well as theta power could also be influenced by methylphenidate when certain doses and duration of treatment is reached. Unfortunately, we are not able to provide estimations on this based on the results of the current study. This issue should be studied further.

Would LRTC serve as a trait or as a state marker? As we described in Introduction, LRTC reflects the adaptability to maintain the balance between stability and flexibility of neuronal assemblies. An imbalance between stability and flexibility could be found in different psychiatric disorders, and this imbalance can be expressed through either increased or reduced LRTC, when compared to a control group in each study. Similar findings could be found in the study of Gärtner et al. [32] in sample of patients with depression, they found changes in LRTC after the intervention, this change associated with improvement in symptomatology. In this sense, LRTC could contain both trait and state aspects. However, in this study, the finding about no change in LRTC after 4-week medication seems to indicate that, the LRTC contains trait characteristic. In contrast, there was also study [66] demonstrated that LRTC changed /reduced after 40-h sleep deprivations. This finding seems to indicate the state aspect of LRTC. Whatsoever, to this issue more research is necessary and worthwhile.

Some limitations of this study need to be mentioned: a lack of control sample in this study prevents us to draw a conclusion on the role of LRTC, especially in regards to alpha and theta oscillations in the pathological mechanisms underlying ADHD and comorbid depressive disorder. We cannot be certain that the observed changes are linked directly to methylphenidate due to the lack of placebo control group. Also, the relatively short duration of treatment increases the difficulty for us to detect the effect; particularly because many patients did not reach the target dose due to side effects [44]. Additionally, the trait and state characteristics of LRTC should be considered during data collection and analyzing. Findings of existing researches supplied vague and inconsistent results; there is both evidence for trait [32] and state [66] aspects of LRTC. Although the current study has taken this issue already into account and kept the first 10 min of the total 15 min resting EEG to reduce the CNS arousal effect, there is so far no uniform standard when LRTC is analyzed. In further studies, these limitations should be considered and probably controlled.

In conclusion, the current results provide direct evidence for the presence of LRTCs in adult patients with ADHD. Different from typical patients in depressive episode in context of a major depression disorder, our remaining depressed aADHD patients showed due to emotional lability and hyperactivity/impulsivity tendencies toward rapid mood changes and moment-to-moment dynamics in behavior. The impact of additional depressive symptoms could be clearly obtained based on comprehensive evaluation about clinical relevant symptoms but potentially in temporal structure quantified by the LRTC in different frequency band of neural oscillations. This study failed to show changes in LRTCs following treatment with extended release methylphenidate. However, our depressed ADHD patients did seem to experience more benefits from the therapy based on self-reports.

## Supplementary Information

Below is the link to the electronic supplementary material.

Supplementary file1 (DOCX 130 KB)

## Data Availability

The data set supporting the conclusions of this article are included within the article. The spreadsheets and corresponding syntax are available on request from the first author.

## References

[1] Fayyad J, Sampson NA, Hwang I, Adamowski T, Aguilar-Gaxiola S, Al-Hamzawi A, Andrade LHSG, Borges G, de Girolamo G, Florescu S, Gureje O, Haro JM, Hu C, Karam EG, Lee S, Navarro-Mateu F, O'Neill S, Pennell B-E, Piazza M, Posada-Villa J, ten Have M, Torres Y, Xavier M, Zaslavsky AM, Kessler RC (2017). The descriptive epidemiology of DSM-IV Adult ADHD in the World Health Organization World Mental Health Surveys. Attent Deficit Hyperact Disord.

[2] Faraone SV, Asherson P, Banaschewski T, Biederman J, Buitelaar JK, Ramos-Quiroga JA, Rohde LA, Sonuga-Barke EJS, Tannock R, Franke B (2015). Attention-deficit/hyperactivity disorder. Nat Rev Dis Primers.

[3] Biederman J, Petty CR, Woodworth KY, Lomedico A, Hyder LL, Faraone SV (2012). Adult outcome of attention-deficit/hyperactivity disorder: a controlled 16-year follow-up study. J Clin Psychiatry.

[4] Jacob CP, Romanos J, Dempfle A, Heine M, Windemuth-Kieselbach C, Kruse A, Reif A, Walitza S, Romanos M, Strobel A, Brocke B, Schäfer H, Schmidtke A, Böning J, Lesch K-P (2007). Co-morbidity of adult attention-deficit/hyperactivity disorder with focus on personality traits and related disorders in a tertiary referral center. Eur Arch Psychiatry Clin Neurosci.

[5] Kessler RC, Adler L, Barkley R, Biederman J, Conners CK, Demler O, Faraone SV, Greenhill LL, Howes MJ, Secnik K, Spencer T, Ustun TB, Walters EE, Zaslavsky AM (2006). The prevalence and correlates of adult ADHD in the United States: results from the National Comorbidity Survey Replication. Am J Psychiatry.

[6] Mayes SD, Calhoun SL, Waxmonsky JG, Kokotovich C, Baweja R, Lockridge R, Bixler EO (2019). Demographic differences in disruptive mood dysregulation disorder symptoms in ADHD, autism, and general population samples. J Atten Disord.

[7] Sobanski E (2006). Psychiatric comorbidity in adults with attention-deficit/hyperactivity disorder (ADHD). Eur Arch Psychiatry Clin Neurosci.

[8] Fischer AG, Bau CHD, Grevet EH, Salgado CAI, Victor MM, Kalil KLS, Sousa NO, Garcia CR, Belmonte-de-Abreu P (2007). The role of comorbid major depressive disorder in the clinical presentation of adult ADHD. J Psychiatr Res.

[9] Halmøy A, Fasmer OB, Gillberg C, Haavik J (2009). Occupational outcome in adult ADHD: impact of symptom profile, comorbid psychiatric problems, and treatment: a cross-sectional study of 414 clinically diagnosed adult ADHD patients. J Atten Disord.

[10] Ramos-Quiroga JA, Montoya A, Kutzelnigg A, Deberdt W, Sobanski E (2013). Attention deficit hyperactivity disorder in the European adult population: prevalence, disease awareness, and treatment guidelines. Curr Med Res Opin.

[11] McIntyre RS, Kennedy SH, Soczynska JK, Nguyen HTT, Bilkey TS, Woldeyohannes HO, Nathanson JA, Joshi S, Cheng JSH, Benson KM, Muzina DJ (2010). Attention-deficit/hyperactivity disorder in adults with bipolar disorder or major depressive disorder: results from the international mood disorders collaborative project. Primary Care Compan J Clin Psychiatry.

[12] Katzman MA, Bilkey TS, Chokka PR, Fallu A, Klassen LJ (2017). Adult ADHD and comorbid disorders: clinical implications of a dimensional approach. BMC Psychiatry.

[13] Thapar A, Cooper M (2016). Attention deficit hyperactivity disorder. The Lancet.

[14] Paucke M, Stibbe T, Huang J, Strauss M (2021). Differentiation of ADHD and depression based on cognitive performance. J Atten Disord.

[15] Huang J, Ulke C, Strauss M (2019). Brain arousal regulation and depressive symptomatology in adults with attention-deficit/hyperactivity disorder (ADHD). BMC Neurosci.

[16] Kim JW, Kim SY, Choi J-W, Kim KM, Nam SH, Min KJ, Lee YS, Choi TY (2017). Differences in resting-state quantitative electroencephalography patterns in attention deficit/hyperactivity disorder with or without comorbid symptoms. Clin Psychopharmacol Neurosci.

[17] Loo SK, Cho A, Hale TS, McGough J, McCracken J, Smalley SL (2013). Characterization of the theta to beta ratio in ADHD: identifying potential sources of heterogeneity. J Atten Disord.

[18] Newson JJ, Thiagarajan TC (2018). EEG frequency bands in psychiatric disorders: a review of resting state studies. Front Hum Neurosci.

[19] Adamou M, Fullen T, Jones SL (2020). EEG for diagnosis of adult ADHD: a systematic review with narrative analysis. Front Psych.

[20] Lenartowicz A, Loo SK (2014). Use of EEG to diagnose ADHD. Curr Psychiatry Rep.

[21] Linkenkaer-Hansen K, Nikouline VV, Palva JM, Ilmoniemi RJ (2001). Long-range temporal correlations and scaling behavior in human brain oscillations. J Neurosci.

[22] Nikulin VV, Brismar T (2004). Long-range temporal correlations in alpha and beta oscillations: effect of arousal level and test-retest reliability. Clin Neurophysiol.

[23] Palva JM, Zhigalov A, Hirvonen J, Korhonen O, Linkenkaer-Hansen K, Palva S (2013). Neuronal long-range temporal correlations and avalanche dynamics are correlated with behavioral scaling laws. Proc Natl Acad Sci USA.

[24] Smit DJA, Linkenkaer-Hansen K, de Geus EJC (2013). Long-range temporal correlations in resting-state α oscillations predict human timing-error dynamics. J Neurosci.

[25] Kello CT, Brown GDA, Ferrer-I-Cancho R, Holden JG, Linkenkaer-Hansen K, Rhodes T, van Orden GC (2010). Scaling laws in cognitive sciences. Trends Cogn Sci.

[26] Sugimura K, Iwasa Y, Kobayashi R, Honda T, Hashimoto J, Kashihara S, Zhu J, Yamamoto K, Kawahara T, Anno M, Nakagawa R, Hatano K, Nakao T (2021). Association between long-range temporal correlations in intrinsic EEG activity and subjective sense of identity. Sci Rep.

[27] Hardstone R, Poil S-S, Schiavone G, Jansen R, Nikulin VV, Mansvelder HD, Linkenkaer-Hansen K (2012). Detrended fluctuation analysis: a scale-free view on neuronal oscillations. Front Physiol.

[28] Herzog ND, Steinfath TP, Tarrasch R (2021). Critical dynamics in spontaneous resting-state oscillations are associated with the attention-related P300 ERP in a Go/Nogo task. Front Neurosci.

[29] Irrmischer M, Poil S-S, Mansvelder HD, Intra FS, Linkenkaer-Hansen K (2018). Strong long-range temporal correlations of beta/gamma oscillations are associated with poor sustained visual attention performance. Eur J Neurosci.

[30] Monto S, Vanhatalo S, Holmes MD, Palva JM (2007) Epileptogenic neocortical networks are revealed by abnormal temporal dynamics in seizure-free subdural EEG. Cerebral cortex (New York, N.Y: 1991) 17(6):1386–1393. 10.1093/cercor/bhl049 10.1093/cercor/bhl049 16908492

[31] Bachmann M, Suhhova A, Lass J, Aadamsoo K, Võhma Ü, Hirnikus H Detrended Fluctuation Analysis of EEG in Depression. In: Roa Romero L (Eds) XIII Mediterranean Conference on Medical and Biological Engineering and Computing 2013, IFMBE Proceedings, vol 41

[32] Gärtner M, Irrmischer M, Winnebeck E, Fissler M, Huntenburg JM, Schroeter TA, Bajbouj M, Linkenkaer-Hansen K, Nikulin VV, Barnhofer T (2017). Aberrant long-range temporal correlations in depression are attenuated after psychological treatment. Front Hum Neurosci.

[33] Montez T, Poil S-S, Jones BF, Manshanden I, Verbunt JPA, van Dijk BW, Brussaard AB, van Ooyen A, Stam CJ, Scheltens P, Linkenkaer-Hansen K (2009). Altered temporal correlations in parietal alpha and prefrontal theta oscillations in early-stage Alzheimer disease. Proc Natl Acad Sci USA.

[34] Nikulin VV, Jönsson EG, Brismar T (2012). Attenuation of long-range temporal correlations in the amplitude dynamics of alpha and beta neuronal oscillations in patients with schizophrenia. Neuroimage.

[35] Hohlefeld FU, Huebl J, Huchzermeyer C, Schneider G-H, Schönecker T, Kühn AA, Curio G, Nikulin VV (2012). Long-range temporal correlations in the subthalamic nucleus of patients with Parkinson's disease. Eur J Neurosci.

[36] Smit DJA, de Geus EJC, van de Nieuwenhuijzen ME, van Beijsterveldt CEM, van Baal GCM, Mansvelder HD, Boomsma DI, Linkenkaer-Hansen K (2011). Scale-free modulation of resting-state neuronal oscillations reflects prolonged brain maturation in humans. J Neurosci.

[37] Kooij JJS, Bijlenga D, Salerno L, Jaeschke R, Bitter I, Balázs J, Thome J, Dom G, Kasper S, Nunes Filipe C, Stes S, Mohr P, Leppämäki S, Casas M, Bobes J, Mccarthy JM, Richarte V, Kjems Philipsen A, Pehlivanidis A, Niemela A, Styr B, Semerci B, Bolea-Alamanac B, Edvinsson D, Baeyens D, Wynchank D, Sobanski E, Philipsen A, McNicholas F, Caci H, Mihailescu I, Manor I, Dobrescu I, Saito T, Krause J, Fayyad J, Ramos-Quiroga JA, Foeken K, Rad F, Adamou M, Ohlmeier M, Fitzgerald M, Gill M, Lensing M, Motavalli Mukaddes N, Brudkiewicz P, Gustafsson P, Tani P, Oswald P, Carpentier PJ, de Rossi P, Delorme R, Markovska Simoska S, Pallanti S, Young S, Bejerot S, Lehtonen T, Kustow J, Müller-Sedgwick U, Hirvikoski T, Pironti V, Ginsberg Y, Félegyházy Z, Garcia-Portilla MP, Asherson P (2019). Updated European Consensus Statement on diagnosis and treatment of adult ADHD. Eur Psychiatry.

[38] Ros T, Frewen P, Théberge J, Michela A, Kluetsch R, Mueller A, Candrian G, Jetly R, Vuilleumier P, Lanius RA (2017) Neurofeedback tunes scale-free dynamics in spontaneous brain activity. Cerebral Cortex (New York, N.Y. : 1991) 27(10):4911–4922. 10.1093/cercor/bhw285 10.1093/cercor/bhw285 27620975

[39] Smith RJ, Sugijoto A, Rismanchi N, Hussain SA, Shrey DW, Lopour BA (2017). Long-range temporal correlations reflect treatment response in the electroencephalogram of patients with infantile spasms. Brain Topogr.

[40] Bornas X, Noguera M, Balle M, Morillas-Romero A, Aguayo-Siquier B, Tortella-Feliu M, Llabrés J (2013). Long-range temporal correlations in resting EEG. J Psychophysiol.

[41] Bornas X, Fiol-Veny A, Balle M, Morillas-Romero A, Tortella-Feliu M (2015). Long range temporal correlations in EEG oscillations of subclinically depressed individuals: their association with brooding and suppression. Cogn Neurodyn.

[42] Lee J-S, Yang B-H, Lee J-H, Choi J-H, Choi I-G, Kim S-B (2007). Detrended fluctuation analysis of resting EEG in depressed outpatients and healthy controls. Clin Neurophysiol.

[43] Linkenkaer-Hansen K, Monto S, Rytsälä H, Suominen K, Isometsä E, Kähkönen S (2005). Breakdown of long-range temporal correlations in theta oscillations in patients with major depressive disorder. J Neurosci.

[44] Strauß M, Petroff D, Huang J, Ulke C, Paucke M, Bogatsch H, Böhme P, Hoffmann K, Reif A, Kittel-Schneider S, Heuser I, Ahlers E, Gallinat J, Schöttle D, Fallgatter A, Ethofer T, Unterecker S, Hegerl U (2021). The "VIP-ADHD trial": does brain arousal have prognostic value for predicting response to psychostimulants in adult ADHD patients?. Eur Neuropsychopharmacol.

[45] Retz-Junginger P, Retz W, Blocher D, Weijers HG, Trott GE, Wender PH, Rössler M (2002) Wender Utah Rating Scale (WURS-k) Die deutsche Kurzform zur retrospektiven Erfassung des hyperkinetischen Syndroms bei Erwachsenen (Wender Utah rating scale. The short-version for the assessment of the attention-deficit hyperactivity disorder in adults). Der Nervenarzt 73(9):830–838. 10.1007/s00115-001-1215-x 10.1007/s00115-001-1215-x 12215873

[46] Christiansen H, Hirsch O, Abdel-Hamid M, Kis B (2014) Conners Skalen zu Aufmerksamkeit und Verhalten für Erwachsene. Deutschsprachige Adaptation der Conners' Adult ADHD Rating Scales (CAARS™) von C. Keith Conners, Drew Erhardt und Elizabeth Sparrow. Hans Huber, Bern

[47] Christiansen H, Hirsch O, Philipsen A, Oades RD, Matthies S, Hebebrand J, Ueckermann J, Abdel-Hamid M, Kraemer M, Wiltfang J, Graf E, Colla M, Sobanski E, Alm B, Rösler M, Jacob C, Jans T, Huss M, Schimmelmann BG, Kis B (2013). German validation of the conners adult ADHD rating scale-self-report: confirmation of factor structure in a large sample of participants with ADHD. J Atten Disord.

[48] Beck A, Steer R, Brown G (1996). Beck Depression Inventory-II.

[49] Montgomery SA, Asberg M (1979). A new depression scale designed to be sensitive to change. British J Psychiatry.

[50] Horowitz L, Strauß B, Thomas A, Kordy H (1994). Inventar zur Erfassung interpersonaler Probleme – Deutsche Version.

[51] Angermeyer M, Kilian R, Matschinger H (2000) WHOQOL-100 und WHOQOL-BREF. Handbuch für die deutschsprachigen Versionen der WHO Instrumente zur Erfassung von Lebensqualität, 1st edn. Hogrefe, Göttingen

[52] Peng CK, Buldyrev SV, Havlin S, Simons M, Stanley HE, Goldberger AL (1994). Mosaic organization of DNA nucleotides. Physical review. E Statist Phys Plasmas Fluids Relat Interdisciplin Topics.

[53] Giedd JN, Blumenthal J, Jeffries NO, Castellanos FX, Liu H, Zijdenbos A, Paus T, Evans AC, Rapoport JL (1999). Brain development during childhood and adolescence: a longitudinal MRI study. Nat Neurosci.

[54] Sowell ER, Peterson BS, Thompson PM, Welcome SE, Henkenius AL, Toga AW (2003). Mapping cortical change across the human life span. Nat Neurosci.

[55] Feinberg I (1982). Schizophrenia: caused by a fault in programmed synaptic elimination during adolescence?. J Psychiatr Res.

[56] McGlashan TH, Hoffman RE (2000). Schizophrenia as a disorder of developmentally reduced synaptic connectivity. Arch Gen Psychiatry.

[57] Tang G, Gudsnuk K, Kuo S-H, Cotrina ML, Rosoklija G, Sosunov A, Sonders MS, Kanter E, Castagna C, Yamamoto A, Yue Z, Arancio O, Peterson BS, Champagne F, Dwork AJ, Goldman J, Sulzer D (2014). Loss of mTOR-dependent macroautophagy causes autistic-like synaptic pruning deficits. Neuron.

[58] Moran JK, Michail G, Heinz A, Keil J, Senkowski D (2019). Long-range temporal correlations in resting state beta oscillations are reduced in schizophrenia. Front Psych.

[59] Jia H, Yu D (2019). Attenuated long-range temporal correlations of electrocortical oscillations in patients with autism spectrum disorder. Dev Cogn Neurosci.

[60] Libutzki B, May M, Gleitz M, Karus M, Neukirch B, Hartman CA, Reif A (2020). Disease burden and direct medical costs of incident adult ADHD: A retrospective longitudinal analysis based on German statutory health insurance claims data. Eur Psychiatry.

[61] Challman TD, Lipsky JJ (2000). Methylphenidate: its pharmacology and uses. Mayo Clin Proc.

[62] Yadid G, Friedman A Dynamics of the dopaminergic system as a key component to the understanding of depression 172:265–286. 10.1016/S0079-6123(08)00913-8 10.1016/S0079-6123(08)00913-8 18772037

[63] Zhigalov A, Kaplan A, Palva JM (2016). Modulation of critical brain dynamics using closed-loop neurofeedback stimulation. Clin Neurophysiol.

[64] Skirrow C, McLoughlin G, Banaschewski T, Brandeis D, Kuntsi J, Asherson P (2015). Normalisation of frontal theta activity following methylphenidate treatment in adult attention-deficit/hyperactivity disorder. Eur neuropsychopharmacol.

[65] Linkenkaer-Hansen K, Smit DJA, Barkil A, van Beijsterveldt TEM, Brussaard AB, Boomsma DI, van Ooyen A, de Geus EJC (2007). Genetic contributions to long-range temporal correlations in ongoing oscillations. J Neurosci.

[66] Meisel C, Bailey K, Achermann P, Plenz D (2017). Decline of long-range temporal correlations in the human brain during sustained wakefulness. Sci Rep.

