# Supplementary material for: The role of comorbid depressive symptoms on long-range temporal correlations in resting EEG in adults with ADHD
Source: Eur Arch Psychiatry Clin Neurosci. 2022 Jul 4;272(8):1421–35. doi: 10.1007/s00406-022-01452-2 (PMC9653316; doi:10.1007/s00406-022-01452-2)
Supplement: Supplementary file 1 — Supplementary file1 (DOCX 130 KB) [file 406_2022_1452_MOESM1_ESM.docx]

**The Role of Comorbid Depressive Symptoms on Long-Range Temporal Correlations in Resting EEG in Adults with ADHD**

# Jue Huang1*, Eike Ahlers2, Holger Bogatsch4, Pierre Böhme5, Thomas Ethofer6,7, Andreas J Fallgatter7, Jürgen Gallinat8, Ulrich Hegerl9, Isabella Heuser2, Knut Hoffmann5, Sarah Kittel-Schneider9,10, Andreas Reif9, Daniel Schöttle8, Stefan Unterecker10, Matti Gärtner2,3†, and Maria Strauß1†

1Department of Psychiatry and Psychotherapy, University of Leipzig, Leipzig, 04103, Germany

2Department of Psychiatry and Psychotherapy, Charité – Universitätsmedizin Berlin, Berlin, 10117, Germany

3MSB Medical School Berlin, Berlin, 14179, Germany

4Clinical Trial Centre Leipzig, Faculty of Medicine, University of Leipzig, Leipzig, 04107, Germany

5Department of Psychiatry Psychotherapy and Preventive Medicine, University Hospital of Bochum, Bochum, 44791, Germany

6Department of Biomedical Magnetic Resonance, University Hospital of Tübingen, Tübingen, 72076, Germany

7Department of Psychiatry and Psychotherapy, Tübingen Center for Mental Health (TüCMH), University of Tübingen, Tübingen, 72076, Germany

8Department of Psychiatry and Psychotherapy, University Medical Center Hamburg-Eppendorf, Hamburg, 20251, Germany

9Department of Psychiatry, Psychotherapy and Psychosomatic Medicine, University Hospital of Frankfurt – Goethe University, Frankfurt am Main, 60528, Germany

10Department of Psychiatry, Psychosomatics and Psychotherapy, University Hospital of Würzburg, Würzburg, 97080, Germany

* Corresponding author. E-Mail: [jue.huang@medizin.uni-leipzig.de](mailto:jue.huang@medizin.uni-leipzig.de) (ORCID: 0000-0003-1830-9340)

Contributing authors: [jue.huang@medizin.uni-leipzig.de](mailto:jue.huang@medizin.uni-leipzig.de); [Eike.Ahlers@charite.de](mailto:Eike.Ahlers@charite.de); [holger.bogatsch@zks.uni-leipzig.de](mailto:holger.bogatsch@zks.uni-leipzig.de); [pierre.boehme@lwl.org](mailto:pierre.boehme@lwl.org); [Thomas.Ethofer@med.uni-tuebingen.de](mailto:Thomas.Ethofer@med.uni-tuebingen.de); [andreas.fallgatter@med.uni-tuebingen.de](mailto:andreas.fallgatter@med.uni-tuebingen.de); [j.gallinat@uke.de](mailto:j.gallinat@uke.de); [ulrich.hegerl@deutsche-depressionshilfe.de](mailto:ulrich.hegerl@deutsche-depressionshilfe.de); [katharina.schmalfeld@charite.de](mailto:katharina.schmalfeld@charite.de); [knut.hoffmann@lwl.org](mailto:knut.hoffmann@lwl.org); [Kittel_S@ukw.de](mailto:Kittel_S@ukw.de); [andreas.reif@kgu.de](mailto:andreas.reif@kgu.de); [d.schoettle@uke.de](mailto:d.schoettle@uke.de); [unterecker_s@ukw.de](mailto:unterecker_s@ukw.de); [matti.gaertner@charite.de](mailto:matti.gaertner@charite.de); [Maria.Strauss@medizin.uni-leipzig.de](mailto:Maria.Strauss@medizin.uni-leipzig.de)

† These authors contributed equally to this work and share senior authorship

**Table S1.** Results of one-sample Kolmogorov-Smirnov Test for normality

|  |  | df | Test Statistic | Significance |
| --- | --- | --- | --- | --- |
| Age |  | 79 | 0.138 | **<0.001** |
| CGI-S |  | 79 | 0.411 | **<0.001** |
| BDI |  | 79 | 0.149 | **<0.001** |
| MADRS |  | 79 | 0.156 | **<0.001** |
| CAARS | DSM-G | 79 | 0.141 | **<0.001** |
|  | DSM-IA | 79 | 0.196 | **<0.001** |
|  | DSM-HYI | 79 | 0.078 | 0.200 |
|  | IA/ME | 79 | 0.164 | **<0.001** |
|  | HY/RE | 79 | 0.093 | 0.086 |
|  | IMP/EL | 79 | 0.080 | 0.200 |
|  | SC | 79 | 0.078 | 0.200 |
|  | ADHS-Index | 79 | 0.081 | 0.200 |
| WURS-K |  | 79 | 0.162 | **<0.001** |
| QoL |  | 79 | 0.074 | 0.200 |
| IIP |  | 79 | 0.099 | 0.052 |
| Mean delta-LRTC |  | 79 | 0.092 | 0.094 |
| Mean theta-LRTC |  | 79 | 0.095 | 0.075 |
| Mean alpha-LRTC |  | 79 | 0.109 | **0.021** |
| Mean beta-LRTC |  | 79 | 0.120 | **0.007** |

Bold fonts indicate statistical significance; statistical significance value for these tests was set at 0.05

CGI-S: Severity Scale of the Clinical Global Impression

BDI: Beck Depression Inventory

MADRS: Montgomery-Asberg Depression Rating Scale

CAARS: the Conners’ Adult ADHD Rating Scale

DSM-G, DSM-IA and DSM-HYI: three subscales of CAARS consisting DSM-IV ADHD symptoms

IA/ME: inattention/memory

HY/RE: hyperactivity/restlessness

IMP/EL: impulsivity/ emotional lability

SC: self-concept

WURS-K: German Wender Utah Rating Scale

QoL: quality of life as measured by German Version of the World Health Organization Quality of Life Questionnaire

IIP: interpersonal problems as measured by German Inventory of Interpersonal Problems


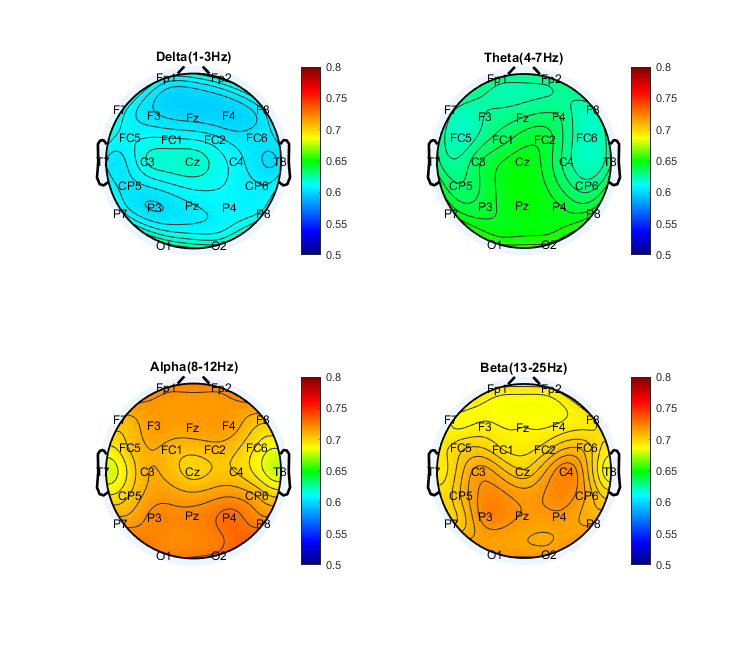


**Figure S1.** Topographical distributions of LRTC in examined frequency bands in all patients with ADHD.

**Table S2.** Descriptive statistics to examine presence of baseline LRTC in corresponding EEG frequency band at each electrode site

| Electrode site | Delta (1-3Hz) |  | Theta (4-7Hz) |  | Alpha (8-12Hz) |  | Beta (13-25Hz) |
| --- | --- | --- | --- | --- | --- | --- | --- |
| Fp1 | 0.62±0.06 |  | 0.63±0.09 |  | 0.73±0.10 |  | 0.70±0.09 |
| Fp2 | 0.62±0.06 |  | 0.63±0.09 |  | 0.73±0.10 |  | 0.70±0.09 |
| F3 | 0.60±0.07 |  | 0.63±0.08 |  | 0.72±0.10 |  | 0.69±0.09 |
| F4 | 0.60±0.07 |  | 0.63±0.09 |  | 0.72±0.10 |  | 0.69±0.09 |
| F7 | 0.62±0.05 |  | 0.63±0.08 |  | 0.71±0.09 |  | 0.69±0.09 |
| F8 | 0.61±0.06 |  | 0.62±0.09 |  | 0.71±0.10 |  | 0.70±0.09 |
| Fz | 0.60±0.07 |  | 0.63±0.10 |  | 0.72±0.10 |  | 0.69±0.10 |
| FC1 | 0.61±0.08 |  | 0.64±0.09 |  | 0.71±0.09 |  | 0.70±0.09 |
| FC2 | 0.61±0.08 |  | 0.64±0.10 |  | 0.71±0.09 |  | 0.70±0.09 |
| FC5 | 0.61±0.06 |  | 0.62±0.07 |  | 0.71±0.09 |  | 0.70±0.10 |
| FC6 | 0.60±0.07 |  | 0.62±0.08 |  | 0.70±0.09 |  | 0.71±0.10 |
| C3 | 0.62±0.07 |  | 0.64±0.09 |  | 0.71±0.09 |  | 0.72±0.09 |
| C4 | 0.61±0.07 |  | 0.63±0.08 |  | 0.71±0.09 |  | 0.73±0.08 |
| T7 | 0.61±0.06 |  | 0.64±0.09 |  | 0.68±0.09 |  | 0.70±0.09 |
| T8 | 0.60±0.05 |  | 0.63±0.09 |  | 0.68±0.08 |  | 0.69±0.08 |
| CP5 | 0.61±0.06 |  | 0.63±0.08 |  | 0.70±0.09 |  | 0.71±0.09 |
| CP6 | 0.61±0.05 |  | 0.63±0.08 |  | 0.72±0.09 |  | 0.72±0.09 |
| P3 | 0.60±0.06 |  | 0.65±0.09 |  | 0.73±0.11 |  | 0.73±0.09 |
| P4 | 0.61±0.06 |  | 0.65±0.08 |  | 0.74±0.10 |  | 0.72±0.09 |
| P7 | 0.61±0.07 |  | 0.65±0.10 |  | 0.72±0.09 |  | 0.70±0.10 |
| P8 | 0.62±0.06 |  | 0.65±0.09 |  | 0.73±0.10 |  | 0.72±0.09 |
| Pz | 0.61±0.06 |  | 0.65±0.10 |  | 0.73±0.10 |  | 0.72±0.09 |
| O1 | 0.63±0.08 |  | 0.66±0.10 |  | 0.73±0.11 |  | 0.72±0.10 |
| O2 | 0.63±0.07 |  | 0.66±0.10 |  | 0.73±0.10 |  | 0.72±0.10 |
| Cz | 0.62±0.08 |  | 0.65±0.10 |  | 0.70±0.09 |  | 0.70±0.09 |

N=85

Entries are mean ± standard deviation

**Table S3.** ADHD+ and ADHD- groups did not differ regarding differences between baseline and final visit for CAARS scores.

|  |  | Test Statistic | Significance |
| --- | --- | --- | --- |
| CAARS | DSM-G | -0.718 | .472 |
|  | DSM-IA | -0.910 | .363 |
|  | DSM-HYI | -0.433 | .665 |
|  | IA/ME | -0.155 | .877 |
|  | HY/RE | -0.576 | .565 |
|  | IMP/EL | -1.183 | .237 |
|  | SC | -0.724 | .469 |
|  | ADHS-Index | -0.427 | .669 |
